# TLR9 activation cooperates with T cell checkpoint blockade to regress poorly immunogenic melanoma

**DOI:** 10.1186/s40425-019-0811-x

**Published:** 2019-11-26

**Authors:** Matthew J. Reilley, Brittany Morrow, Casey R. Ager, Arthur Liu, David S. Hong, Michael A. Curran

**Affiliations:** 1 Department of Medicine, The University of Virginia, Charlottesville, VA 22903 USA; 2 Department of Immunology, The University of Texas MD Anderson Cancer Center, Houston, TX 77030 USA; 3 Department of Investigational Cancer Therapeutics, The University of Texas MD Anderson Cancer Center, Houston, TX 77030 USA; 4 The University of Texas MD Anderson UTHealth Graduate School of Biomedical Sciences; Immunology Program, Houston, TX 77030 USA

**Keywords:** TLR9, CTLA-4, PD-1, Immunotherapy, MGN1703

## Abstract

Tumors that lack pre-existing immune infiltration respond poorly to T cell checkpoint blockade immunotherapy. These cancers often surround themselves with high densities of suppressive myeloid stroma while excluding immunostimulatory dendritic cells. Tumor-resident myeloid cells and selected lymphocyte populations retain expression of Toll-like Receptors (TLR) that sense common features of pathogens and activate innate immunity in response. We explored whether agonists of TLR9 could augment innate immunity to promote tumor regression alone or in combination with T cell checkpoint blockade. In the setting of the immunogenic B16-Ova (Ovalbumin) expressing melanoma model, local injection of the CpG oligonucleotide TLR9 agonist ODN1826 combined with systemic CTLA-4 blockade cured 45% of mice of both their treated and an untreated tumor on the opposite flank demonstrating the synergistic potential of this combination. Next, in the non-immunogenic B16-F10 melanoma model, we showed that only intra-tumoral, but not systemic TLR9 activation augments the therapeutic potential of checkpoint blockade. In this setting, intra-tumoral TLR9 activation cooperated equally with either CTLA-4 or PD-1 blockade co-administered locally or given systemically; however, the uninjected tumor rarely regressed. Anti-CTLA-4 combinations were associated with improved intra-tumoral CD8 to regulatory T cell ratios, while anti-PD-1 combinations elicited improved ratios of CD8 T cells relative to suppressive myeloid stroma. Using both a TLR9 agonist (MGN1703) and a CTLA-4 antibody (9D9-IgG2a) of increased potency cured 50% of bi-lateral B16-F10 melanoma. These findings suggest that intra-tumoral TLR9 agonists can improve sensitivity of poorly immunogenic tumors to T cell checkpoint blockade, and that newer, higher potency TLR agonists and checkpoint antibodies can raise the therapeutic ceiling for this combination therapy.

## Introduction

Tumors actively condition their microenvironments to foster recruitment of suppressive myeloid stroma and dampen accumulation of potentially immunostimulatory antigen-presenting cells such as dendritic cells. Lack of pro-inflammatory myeloid cells fosters immune ignorance of the tumor as a result of insufficient tumor antigen cross-presentation. Further, the predominant M2 macrophage and myeloid-derived suppressor cell (MDSC) composition of the myeloid stroma effectively shields the tumor from any adaptive immune effectors which do become mobilized. In this setting, blockade of T cell immune checkpoint receptors is often insufficient to mediate any significant regression of cancer.

Toll-like receptors (TLR) sense common features of pathogens and, in response, trigger innate immune activation including secretion of type I Interferons. Provision of toll-like receptor ligands has the potential to reactivate tumor stroma, particularly myeloid cells and B cells, thus increasing both tumor antigen cross-presentation and pro-inflammatory cytokine production [1]. These direct effects on innate immune activation, in turn, foster enhanced activation of adaptive immune effectors (i.e. T and NK cells) increasing both baseline tumor immune infiltration as well as sensitivity to T cell checkpoint blockade therapy.

Agonists of Toll-like receptor 9 (TLR9), which recognizes DNA with unmethylated CpG motifs, can activate B cells, myeloid dendritic cells, and plasmacytoid dendritic cells [2]. Prior publications have demonstrated the potential of various TLR9 agonists administered via intra-tumor injection to augment anti-tumor immunity alone or in combination with T cell checkpoint blocking or T cell co-stimulatory agonist antibodies [3–8]. Despite this, the optimal route of administration for TLR9 agonists, as well as their compatibility with current FDA-approved checkpoint blockade antibodies remains unknown. Further, synthetic TLR9 agonists with enhanced potency relative to classical oligodeoxynucleotide (ODN) agonists have been developed; however, whether the in vitro potency of these drugs translates to enhanced in vivo immunotherapeutic potential has yet to be determined.

Here we show that intra-tumoral administration of the TLR9 agonist ODN1826 [9] synergizes with CTLA-4 blockade to promote rejection of bi-laterally implanted B16-Ovalbumin (B16-Ova) melanoma. As innate agonists of both TLR and the Stimulator of Interferon Genes pathways are now being administered to patients both intra-tumorally as well as systemically, we investigated the impact of route of delivery on the efficacy of ODN1826 with or without anti-CTLA-4 or anti-PD-1 on the progression of bi-laterally implanted B16-F10 parental melanoma. While intra-tumoral ODN1826 benefitted from being combined with either CTLA-4 or PD-1 blocking antibodies, whether they were given systemically (most effective) or locally (less effective), systemic administration of TLR9 agonist showed no efficacy alone or in combination with checkpoint blockade. Mechanistically, the addition of checkpoint blockade improves intratumoral ratios of CD8 T cells relative to suppressive stroma in the uninjected lesion and improves functional attributes of these critical effectors of anti-tumor immunity. Finally, we show that by combining both an enhanced potency TLR9 agonist (MGN1703 [10]) and a depletion-optimized CTLA-4 antibody (9D9-mIgG2a [11]), half of pre-implanted parental B16-F10 melanoma can be cured.

## Materials and methods

### Animals

Male (6wk) C57BL/6 mice were purchased from the Jackson Laboratory (Bar Harbor, ME). All procedures were conducted in accordance with the guidelines established by the U.T. MD Anderson Cancer Center Institutional Animal Care and Use Committee.

### Cell lines and reagents

B16-F10 melanoma and B16-Ova were obtained/created and cultured as described [12, 13].

### Therapeutic antibodies

CTLA-4 (9H10 [Syrian Hamster Ig], 100 μg/dose) and PD-1 (RMP1–14 [Rat IgG2a], 250 μg/dose) antibodies were purchased from BioXCell or Leinco. CTLA-4 (9D9 [Mouse IgG2a], 100μg/dose) was produced by ATUM.

### TLR9 agonists

ODN1826 was obtained from Invivogen, reconstituted in PBS and given either via intra-tumoral (local, 10μg or 30μg in 50ul) or intra-peritoneal (systemic, 10μg or 30μg in 100ul) administration. MGN1703 was obtained from Mologen, diluted in PBS, and administered intra-tumorally at 30μg in 50ul.

### Tumor therapy

Mice were implanted s.c. with 1.5X10^5^ B16-Ova or 2.5X10^4^ B16-F10 cells on the flank as described [12, 13]. For isolation of tumor infiltrating lymphocytes, tumors are implanted in 30% Matrigel (Corning). On days 3, 6, and 9 mice received the indicated antibody and/or TLR9 agonist i.p or intra-tumorally as indicated. B16 melanoma tumors leave a black spot at the injection site which is used to localize intra-tumoral injections when tumors are not yet palpable. Animals are followed and tumor growth tracked until tumors reach 1000mm^3^ in size.

### Cell isolation

Tumors were isolated, digested into single cell suspensions, and enriched for viable lymphocytes as previously described [14, 15].

### Flow cytometry analysis

Tumor infiltrating lymphocytes were isolated by enzymatic digestion of tumor and enrichment over a Histopaque 1119 (Sigma) gradient. Samples were fixed using the Foxp3/Transcription Factor Staining Buffer Set (Thermo) and then stained with up to 12 antibodies at a time from Biolegend, BD Biosciences, and Thermo. Flow cytometry data was collected on an 18-color BD LSR II cytometer and analyzed in FlowJo (Treestar).

### Statistical analysis

All statistics were calculated using Graphpad Prism Version 8 for Windows. Statistical significance was determined using the Mantel-Cox (Logrank) test for survival and ANOVA for tumor-infiltrating lymphocyte analysis. Graphs show mean ± standard deviation unless otherwise indicated. *P*-values less than 0.05 were considered significant.

## Results

### The combination of intra-tumoral ODN1826 and the anti-CTLA-4 antibody 9H10 promotes rejection of bilateral B16-ova melanoma

**Fig. 1:**
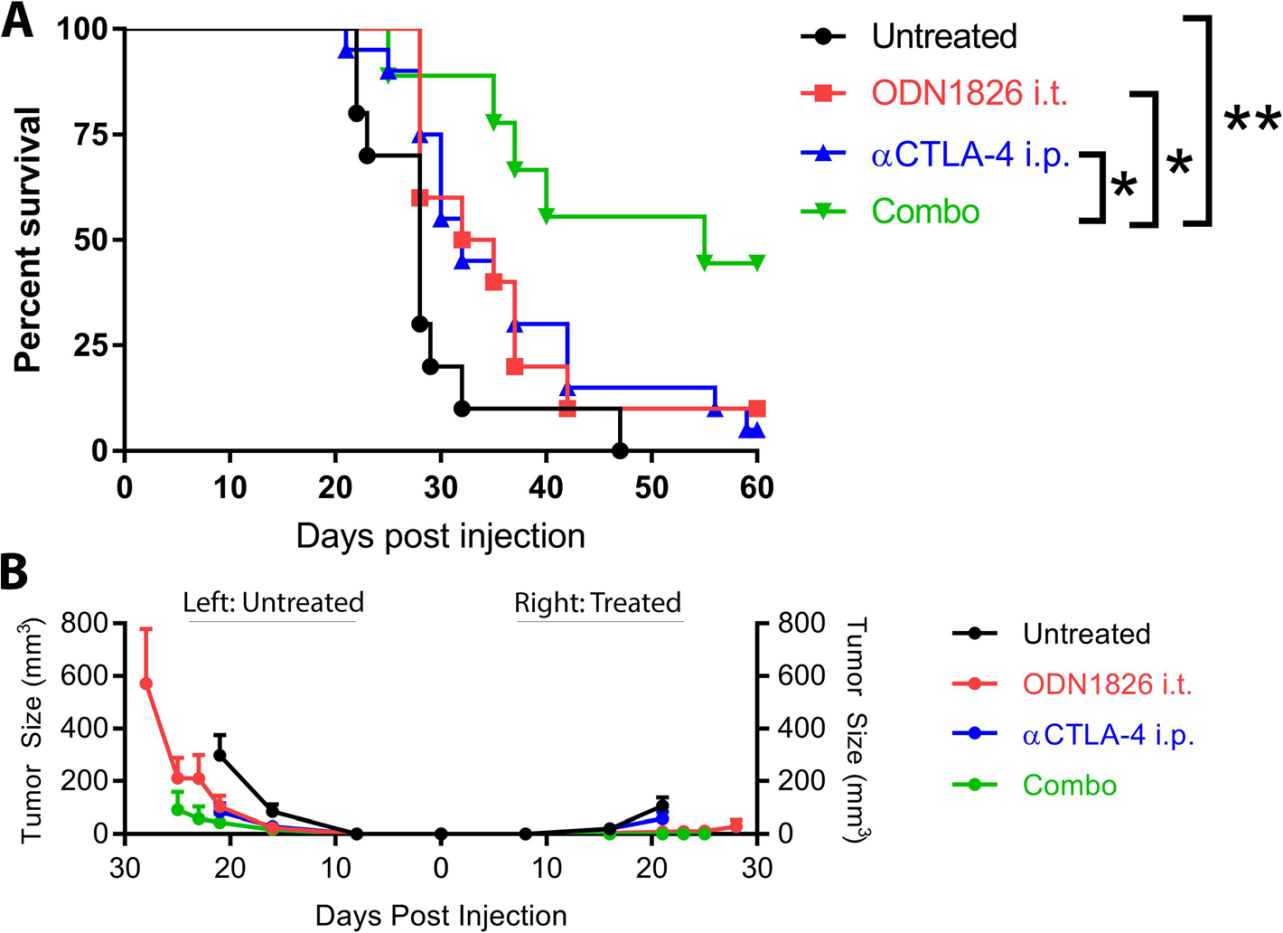
Combination therapy with intra-tumoral ODN1826 and systemic CTLA-4 blockade cures bilateral B16-Ova melanoma. (**a**) C57BL/6 J mice were injected with 1.5 × 10^5^ B16-Ova melanoma cells on the right and left flanks. The right flank tumor was then injected with 30μg of the TLR9 agonist ODN1826 or PBS in 50ul on days 3, 6 and 9 with or without concordant injection of 100μg of the CTLA-4 blocking antibody 9H10 i.p. Survival was monitored and mice were euthanized when tumors reached 1000mm^3^ on either flank. (**b**) Tumor growth was measured with calipers for the right (ODN1826 injected) and left (untreated) tumors and is plotted until the point at which any mice in the group died or their tumors on either flank reached 1000mm^3^. These data represent two experiments of 5 mice per group, all mice shown. Statistical significance was calculated using the log-rank (Mantel-Cox) test. *P < 0.05, **P < 0.01, ***P < 0.001, ****P < 0.0001

We sought to test whether activation of TLR9 through intra-tumoral injection in the B16-Ova melanoma model could potentiate systemic, sterilizing anti-tumor immunity in conjunction with blockade of the T cell immune checkpoint receptor CTLA-4. C57BL/6 J mice were injected with 1.5 × 10^5^ B16-Ova melanoma cells on the right and left flanks. The right flank tumor was then injected with 30μg of the TLR9 agonist ODN1826 or PBS on days 3, 6 and 9 with or without concordant injection of 100μg of the CTLA-4 blocking antibody 9H10 systemically. The combination of TLR9 activation and CTLA-4 blockade cures 44% of mice of both the injected and uninjected tumors, while survival with either monotherapy is 10% or less (Fig. 1a). TLR9 injected tumors on the right flank largely resolve; however, resolution of the left flank tumor is only pronounced in combination with CTLA-4 blockade (Fig. 1b). These data illustrate therapeutic synergy of innate activation of the tumor stroma by the TLR9 agonist combined with regulatory T cell (Treg) depletion and effector T cell checkpoint blockade from the CTLA-4 antibody 9H10. In this setting, the TLR9 agonist converts the injected tumor into an in situ vaccine and the checkpoint provides the conditioning of uninjected tumor sites and protection from attenuation necessary for the mobilized T cells to mediate effective abscopal responses. While this data demonstrates the high therapeutic potential of this combination in an immunogenic tumor setting, we sought to determine the optimal dose, schedule, and administration of this pair in the parental, poorly immunogenic B16-F10 model.

### Intra-tumoral, but not systemic ODN1826 cooperates with either systemic CTLA-4 or PD-1 blockade to treat B16-F10 melanoma

**Fig. 2:**
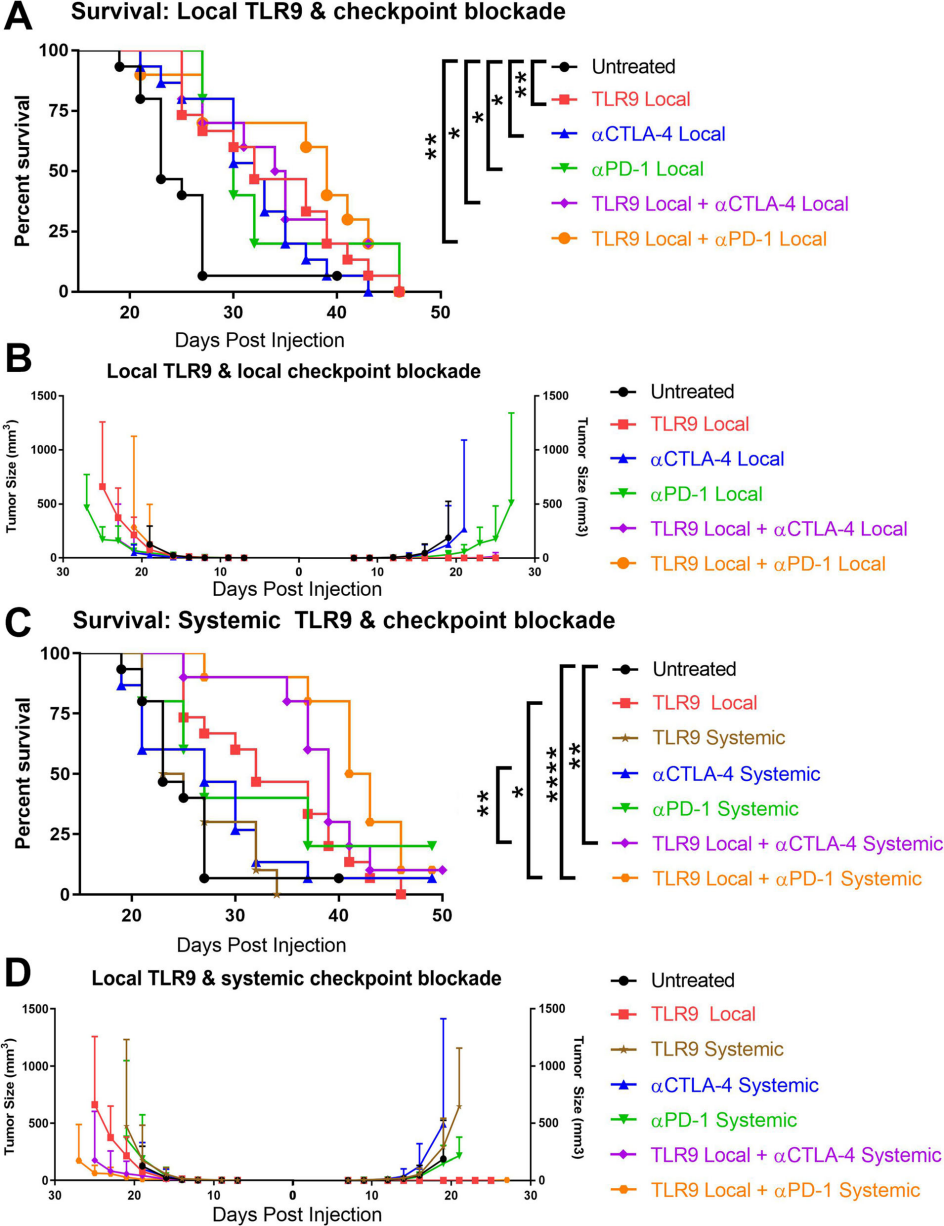
Local but not systemic TLR9 activation cooperates with either CTLA-4 or PD-1 blockade to treat B16-F10 melanoma. C57BL/6 J mice were injected with 2.5 × 10^4^ B16-F10 melanoma cells on the right and left flanks. (**a**) The right flank tumor was then injected with 30μg of the TLR9 agonist ODN1826 or PBS in 50ul on days 3, 6 and 9 or mice received injection of 10μg of the CTLA-4 blocking antibody 9H10 or 10μg of the PD-1 antibody RMP1–14 intra-tumorally. Survival was monitored and mice were euthanized when tumors reached 1000mm^3^. (**b**) Tumor growth was measured with calipers for the right (ODN1826 injected) and left (untreated) tumors. (**c**) As above except that mice received injection of 100μg of the CTLA-4 blocking antibody 9H10 or 250μg of the PD-1 antibody RMP1–14 i.p. Survival was monitored and mice were euthanized when tumors reached 1000mm^3^. (**d**) Tumor growth was measured with calipers for the right (ODN1826 injected) and left (untreated) tumors. These data represent one (antibody monotherapies) to two (all combinations) independent experiments of 5–10 mice per group, all mice are shown. Statistical significance was calculated using the log-rank (Mantel-Cox) test. *P < 0.05, **P < 0.01, ***P < 0.001, ****P < 0.0001

Having shown the cooperative potential of CTLA-4 blockade and TLR9 activation, we sought to determine whether anti-PD-1 could substitute for anti-CTLA-4, whether TLR9 activation was most efficient locally or systemically, and whether both the checkpoint antibody and TLR9 agonist could be given locally to avoid systemic toxicities. We implanted 2.5 × 10^4^ B16-F10 melanoma cells on the right and left flanks and treated mice on days 3, 6, and 9 with ODN1826 at 30μg either intra-tumorally or systemically with or without concomitant anti-CTLA-4 (9H10) or anti-PD-1 either locally (10μg) or systemically (100μg/250μg). Compared with B16-Ova, the non-immunogenic B16-F10 melanoma was significantly less responsive to monotherapy treatments; however, TLR9 agonist (*p* = 0.0054), anti-CTLA-4 (*p* = 0.0125) and anti-PD-1 (*p* = 0.0283) all showed a modest capacity to extend survival (Fig. 2a). Combinations of ODN1826 with local anti-CTLA-4 (*p* = 0.014) or anti-PD-1 (*p* = 0.0053) was superior to control, but failed to show significant improvement versus the component therapies. Intra-tumoral TLR9 agonist readily elicited rejection of the injected lesion; however, there was no evidence of abscopal activity against the uninjected tumor on the opposite flank (Fig. 2b). Given the lack of potentiation of local TLR9 agonist activity by local checkpoint blockade against this poorly immunogenic melanoma, we explored the potential of systemic administration of these immunotherapies.

Systemic ODN1826, CTLA-4 blockade and PD-1 blockade were ineffective at extending survival in bilateral B16-F10 bearing animals. Both the TLR9 agonist and anti-CTLA-4 antibody lost all efficacy when administered systemically, in contrast to their activity when used locally in the right flank lesion (Fig. 2c, d). Given the lack of efficacy of systemic TLR9 agonist, we focused on combinations of local TLR9 agonist and systemic checkpoint blockade. In this context, intra-tumoral ODN1826 combined with systemic CTLA-4 blockade to extend survival versus control (*p* = 0.0016) and versus anti-CTLA-4 alone (*p* = 0.0094). Local TLR9 agonist with systemic PD-1 blockade also extended survival versus control (*p* = 0.0061), but only trended toward superiority to anti-PD-1 alone (*p* = 0.06, Gehan-Breslow-Wilcoxon test). Of note, this combination of local ODN1826 with systemic PD-1 blockade was also superior to local TLR9 agonist (*p* = 0.011); however, the benefit of CTLA-4 blockade and TLR9 agonist over local ODN1826 alone did not reach significance (Fig. 2c, d). In order to understand the mechanisms underlying the differential efficacy across these combinations, we decided to assess each of their impacts on the lymphocyte infiltrate of the uninjected tumor.

### TLR9 activation with CTLA-4 or PD-1 blockade improves ratios of CD8 T cells versus suppressive lymphocytes and myeloid cells

**Fig. 3:**
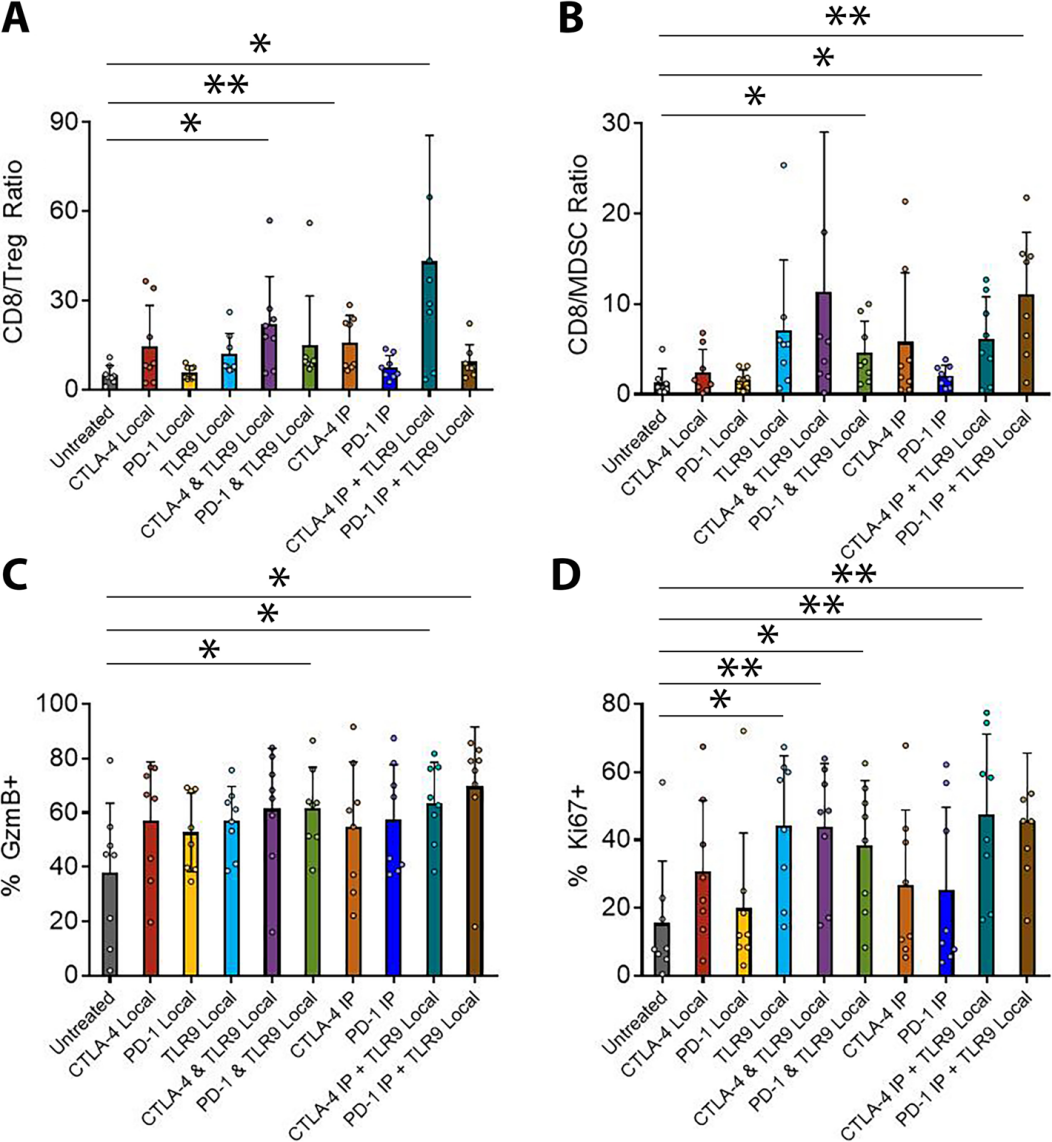
CTLA-4 and PD-1 blockade each potentiate intra-tumoral TLR9 activation through distinct mechanisms. (A) C57BL/6 J mice were injected with 1.5 × 10^5^ B16-Ova melanoma cells on the right and left flanks. The right flank tumor was then injected with 30μg of the TLR9 agonist ODN1826 or PBS in 50ul on days 3, 6 and 9 with or without concordant injection of 100μg anti-CTLA-4 9H10 i.p. or 250μg of anti-PD-1 RMP1–14 i.p. or 10μg of either antibody intra-tumorally. Mice were euthanized on day 14, tumors treated with Collagenase H (Sigma) and DNase (Roche) to produce single cell suspensions, and tumor infiltrating lymphocytes enriched via separation over a Histopaque 1119 (Sigma) density gradient (*n* = 1 experiment with 8 mice / group). Cells were fixed with the FoxP3 fixation kit (Thermo), stained with antibodies and analyzed by flow cytometry. (**a**) The ratios of intra-tumoral CD8 T cells versus FoxP3+ Treg and (**b**) versus CD11b^+^GR-1^+^ MDSC were determined. (**c**) For intra-tumoral CD8 T cells, the percentage expressing Granzyme B and (**d**) Ki67 was also measured. Statistical significance was calculated using the student’s t-test. *P < 0.05, **P < 0.01, ***P < 0.001, ****P < 0.0001

While the intra-tumorally injected melanoma on the right flank almost always resolves, we wanted to assess changes in the immune infiltration of the unmanipulated left flank tumor to measure abscopal potential of each therapy. We established and treated bilateral B16-F10 tumors as described previously except that tumors were implanted in 30% Matrigel (Corning) to facilitate recovery of infiltrating lymphocytes. At day 14 post-implantation, tumors were isolate, dispersed into single cells, enriched for viable lymphocytes over a Histopaque 1119 (Sigma) gradient, and then stained for analysis by flow cytometry. Intra-tumoral ratios of CD8 T cells versus FoxP3^+^ Tregs were significantly increased by the systemic CTLA-4 blockade alone or with intra-tumoral TLR9 agonist consistent with the known activity of this CTLA-4 antibody to deplete Tregs (Fig. 3a and Additional file 1: Figure S1). Despite this, the systemic CTLA-4 blockade and local TLR9 agonist combination trends toward significance over TLR9 alone (*p* = 0.059) and systemic anti-CTLA-4 (*p* = 0.089) but did not reach significance. Notably, the combination of local TLR9 and local CTLA-4 also significantly improved CD8 to Treg ratios in the untreated lesion reflecting improved mobilization of CD8s at the injected lesion, which then traffic to the left flank tumor. We observed little benefit of the PD-1 combination in this regard. In contrast, ratios of CD8 T cells to myeloid-derived suppressor cells (MDSC) benefitted most from the combination of TLR9 agonist delivered to the right flank tumor combined with systemic PD-1 blockade (Fig. 3b). This combination was superior to anti-PD-1 alone; however, not in comparison to local TLR9 agonist alone. Again, local PD-1 blockade combined with TLR9 agonist also significantly improved the CD8 to MDSC ratio in the untreated lesion. There was also a significant improvement in the group receiving systemic CTLA-4 blockade with TLR9 intra-tumorally perhaps reflecting more efficient CD8 mobilization from the untreated tumor Across the analysis of these tumor infiltrating lymphocyte ratios, it was generally only the combinations of local TLR9 agonist and checkpoint blockade which showed significant benefit over untreated. We speculate that the more limited capacity to show statistically relevant benefit over the component monotherapies was likely a product of the limited overall efficacy of these interventions against the parental B16 melanoma.

Consistent with the known of effect of PD-1 blockade to restore suppressed T cell effector function, the most significant elevation of CD8 T cell Granzyme B frequency was observed in mice receiving the combination of local TLR9 agonist and systemic PD-1 blockade (Fig. 3c). Also, local PD-1 blockade and local TLR9 agonist significantly improved Granzyme B, as did systemic CTLA-4 blockade and local ODN1826. As for the effector to suppressor ratios, the combination therapies did not elevate Granzyme B frequency significantly beyond the monotherapies; however, none of the monotherapies conferred significant benefit versus untreated. In terms of proliferation, TLR9 agonist clearly promoted expansion of T cells which trafficked to the untreated lesion (Fig. 3d).

In summary, analysis of the infiltrate of the uninjected melanoma reveals that intra-tumoral TLR9 agonist therapy may mobilize CD8 T cells that traffic to the distal lesion and benefit from anti-CTLA-4 mediated Treg depletion or enhanced expansion relative to suppressive myeloid stroma mediated by anti-PD-1. Although local checkpoint blockade with local ODN1826 failed to statistically improve overall survival versus the component monotherapies, it did improve the immune infiltrate in the distal tumor, albeit to a lesser degree than systemic checkpoint blockade. CD8 T cell effector function was most significantly improved by the combination of local TLR9 activation and PD-1 blockade in a setting where none of the component monotherapies impacted Granzyme B frequency. Local TLR9 agonist alone or in combination with either checkpoint-blocking antibody significantly increased frequencies of actively proliferating CD8 T cells that trafficked to the untreated lesion on the opposite flank. Given these observations, we sought to determine whether a more potent TLR9 agonist and a more efficient Treg depleting CTLA-4 antibody could further augment the efficacy of this combination therapy.

### Intra-tumoral MGN1703 combined with systemic 9D9-IgG2a anti-CTLA-4 antibody cures poorly immunogenic B16-F10 melanoma

**Fig. 4:**
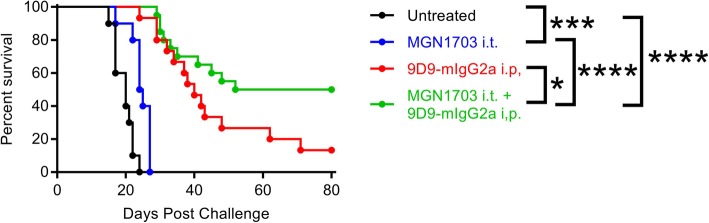
Intra-tumoral MGN1703 combined with systemic 9D9-IgG2a anti-CTLA-4 antibody cures poorly immunogenic B16-F10 melanoma. C57BL/6 J mice were injected with 2.5 × 10^4^ B16-F10 melanoma cells on the right and left flanks. The right flank tumor was then injected with 30μg of the TLR9 agonist MGN1703 or PBS in 50ul on days 3, 6 and 9 with or without concordant injection of 100μg of the CTLA-4 blocking antibody 9D9-mIgG2a i.p. Survival was monitored and mice were euthanized when tumors reached 1000mm^3^ (*n* = 2 independent experiments of 5–10 mice / group, all shown). Statistical significance was calculated using the log-rank (Mantel-Cox) test. *P < 0.05, **P < 0.01, ***P < 0.001, ****P < 0.0001

MGN1703 is a potent, clinical-stage TLR9 agonist which has recently been described [10]. The mouse anti-mouse CTLA-4 antibody 9D9 is an exceptionally efficient depleter of Tregs when expressed with the mouse IgG2a isotype, and is not subject to antibody-mediated neutralization over repeated administration as is the hamster-derived 9H10 clone used previously [11]. C57BL/6 J mice were injected with 2.5 × 10^4^ B16-F10 melanoma cells on the right and left flanks. The right flank tumor was then injected with 30μg of the TLR9 agonist MGN1703 or PBS on days 3, 6 and 9 with or without concordant injection of 100μg of the CTLA-4 antibody 9D9-mIgG2a systemically. Whereas no animals survived in the prior study with ODN1826 and 9H10, 50% of MGN1703 and 9D9-mIgG2a treated mice are cured of bilateral B16-F10 (Fig. 4). The higher activity CTLA-4 antibody accounts for much of this improvement with 13% survival as a monotherapy; however, there appears to be clear synergy with the more potent TLR9 agonist as well (*p* = 0.04). While the improved potency of MGN1703 for TLR9 activation has been reported previously [10], it is impossible for us to preclude a similar benefit with the ODN1826 combination in this context. These data demonstrate that highly significant improvements in survival and cure rate for bilateral melanoma can be achieved by using higher potency TLR9 and CTLA-4 blocking / depleting antibodies.

## Discussion

In this study, we sought to answer a series of questions designed to inform optimal design of the growing number of clinical trials seeking to combine activation of innate immunity through TLR engagement with augmentation of the mobilized T cell response through checkpoint antibody administration. Administration of the TLR9 agonist ODN1826 was consistently effective at inducing rejection of the injected lesion and in creating advantageous CD8 to MDSC rations in distal lesions when given intra-tumorally. In contrast, there was no therapeutic benefit to systemic TLR9 agonist alone or in combination with checkpoint blockade likely reflecting a lack of specific immune activation in the tumor microenvironment. Both CTLA-4 and PD-1 blockade could potentiate in situ vaccination through intra-tumoral TLR9 activation with systemic administration, while local administration only effected sub-therapeutic improvements to the uninjected tumor. In the clinic, local administration would engender no appreciable systemic toxicity and prior publications have suggested that intra-tumoral administration of high order combinations of checkpoint antagonists and co-stimulatory agonists may be significantly more effective than the results we obtained here with monotherapy combinations [15].

Mechanistically, improved CD8 to Treg ratios in the uninjected tumor appeared critical for conditioning an environment in which T cell mobilized from the TLR9-treated lesion could flourish. Although current clinical CTLA-4 antibodies do not efficiently deplete Tregs from solid tumors, development of 2nd generation CTLA-4 antibodies for patients that can kill Tregs either systemically or specifically in the tumor microenvironment are nearing the clinic. Our data would suggest that such drugs may powerfully synergize with TLR agonists to more effectively potentiate abscopal anti-tumor immunity. PD-1 blockade, which is the prevalent immunotherapy in the clinic, also showed equivalent combination potential to CTLA-4 blockade when paired with intra-tumoral TLR9 agonist. Interestingly, the CTLA-4 combination created enhanced CD8 to Treg ratios in the distal tumor, whereas the PD-1 combination yielded improved ratios of CD8 relative to suppressive myeloid stroma. This suggests that the combination of CTLA-4 and PD-1 blockade in this context may synergize in improving therapeutic outcomes in this setting.

It remained unclear to what extent the potency of the component TLR9 agonist and CTLA-4 antibody acted together to set a threshold of efficacy for this combination therapy. We show that a more potent TLR9 agonist (i.e. MGN1703 versus ODN1826) combined with a more potent CTLA-4 antibody (i.e. 9D9-mIgG2a versus 9H10) very significantly increased the therapeutic potential of this combination against bilateral, poorly immunogenic B16-F10 melanoma (0% versus 50% tumor-free survival). In the clinic this suggests that more powerful innate agonists can deliver greater therapeutic benefit and that the advent of anti-human CTLA-4 antibodies with the capacity to deplete Tregs, particularly in a tumor-selective fashion, could profoundly improve outcomes against poorly immunogenic cancers.

## Supplementary information

**Additional file 1.** Flow cytometry gating strategy for tumor infiltrating lymphocyte analysis.

## Data Availability

The datasets used and/or analyzed during the current study available from the corresponding author on request.

## Abbreviations

CTLA-4: Cytotoxic T-lymphocyte–associated antigen 4
MDSC: Myeloid-derived suppressor cell
ODN: Oligodeoxynucleotide
Ova: Ovalbumin
PD-1: Programmed Cell Death 1
TAM: Tumor associated macrophage
TLR: Toll-like Receptor
