# Supplementary material for: TLR9 activation cooperates with T cell checkpoint blockade to regress poorly immunogenic melanoma
Source: J Immunother Cancer. 2019 Nov 26;7:323. doi: 10.1186/s40425-019-0811-x (PMC6880482; doi:10.1186/s40425-019-0811-x)
Supplement: Supplementary file 1 — Additional file 1. Flow cytometry gating strategy for tumor infiltrating lymphocyte analysis. [file 40425_2019_811_MOESM1_ESM.docx]

**Supplemental Figure 1**


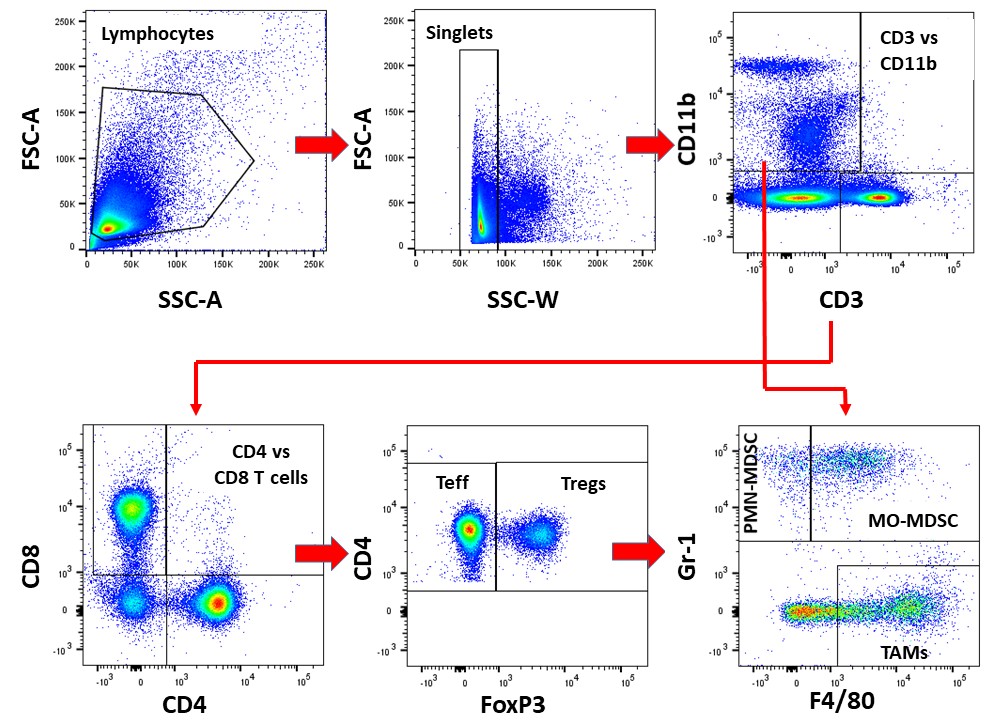


cytometry data was collected on a BD Fortessa Dual cytometer, compensated in FlowJo 7.65 and gated and analyzed in FlowJo 10.5. Representative data is shown to illustrate profiling of major T cell and myeloid populations from B16-F10 tumors.
